# Analysis of flavor characteristics of peanut porridge using gas chromatography-ion mobility spectrometry combined with intelligent sensory technology

**DOI:** 10.3389/fnut.2025.1609333

**Published:** 2025-10-01

**Authors:** Bihua Yuan, Yun Tian, Xuan Zhu, Kaiqi Cheng, Wengang Jin, Qing Liu, Jing Li, Haiyan Sun

**Affiliations:** School of Biological Science and Engineering, Shaanxi University of Technology, Hanzhong, China

**Keywords:** gas chromatography-ion mobility spectrometry, peanut porridge, electronic tongue, electronic nose, flavor characteristics

## Abstract

**Introduction:**

This study aimed to investigate the differences in flavor compounds between aged and fresh peanuts and their effects on peanut porridge aroma, taste, and volatile fingerprint profiles.

**Methods:**

Gas chromatography–ion mobility spectrometry (GC-IMS), combined with electronic sensory technologies such as electronic tongue and electronic nose, was applied to analyze peanut porridge processed under different treatments. Partial least squares-discriminant analysis (PLS-DA) and principal component analysis (PCA) were used to identify characteristic volatiles and discriminate treatments.

**Results:**

Electronic tongue and electronic nose analyses effectively distinguished peanut porridges based on flavor characteristics. A total of 47 volatile compounds were detected, including 10 alcohols, 10 esters, 6 ketones, 5 acids, 2 alkenes, 6 aldehydes, and 8 other compounds. PLS-DA identified 16 characteristic volatiles (VIP > 1), such as 2-pentanone, ethyl hexanoate, 2-acetylfuran, butanal, pentyl acetate, and heptanal. PCA showed that two principal components accounted for 66.7% of the total variance, enabling clear discrimination among treatments.

**Discussion:**

The study systematically explored key differences in volatile compounds between aged and fresh peanuts and analyzed their impact on sensory attributes, particularly aroma and flavor perception. These findings enhance understanding of flavor formation mechanisms in peanut-based products and provide scientific evidence for flavor modulation, formulation optimization, product innovation, and quality control.

## 1 Introduction

Peanut, also known as groundnut, ranks second in production among leguminous crops worldwide (1) and is often referred to as the “longevity nut” (2). China is the world’s largest producer of peanuts, and peanuts serve as a significant economic crop in the country. They are a crucial raw material for producing peanut oil, which leads in total production among China’s oil crops and is considered a high-quality edible oil (3). Peanuts possess high nutritional value, containing various essential amino acids, unsaturated fatty acids, crude fiber, and carotenoids (4). They are rich in quality proteins (5), including eight essential amino acids for humans, which are precursors to peanut flavor compounds (6). The peanut meal produced after oil extraction contains abundant proteins (7), qualifying as high-quality plant protein. Peanuts are also rich in polyphenols and flavonoids (8), earning them the reputation of “green milk” and “plant meat” (9). Additionally, peanuts have been associated with anti-aging, cognitive enhancement, and tumor prevention properties (10). Due to their nutritional value and unique aroma, peanuts are favored by consumers.

Peanuts can be categorized into aged and fresh types. Fresh peanuts have a sweet and crisp texture with a fresh and natural aroma, retaining more of the original flavor of the raw material. They are suitable for foods emphasizing a refreshing taste, such as cold dishes, salad ingredients, peanut milk beverages, ready-to-eat fresh peanuts, and lightly seasoned snacks. Aged peanuts possess a complex and rich aroma with roasted, caramel, and nutty flavors, making them suitable for processing methods requiring deep flavor presentation, such as peanut butter, baked pastries, braised peanuts, peanut brittle, and peanut porridge.

Peanut porridge is a renowned snack in Hanzhong City, highly favored by locals. Unlike other regions where peanut porridge typically involves cooking whole peanut kernels, Hanzhong’s version involves blending peanuts into a slurry to make the porridge, hence also known as peanut slurry porridge or peanut paste porridge. This distinctive preparation method has made it a celebrated local delicacy, praised by visitors. Given peanuts’ rich nutritional profile and their moniker as the “artery cleaner” (10), peanut porridge offers a slightly sweet taste with a strong peanut aroma and soft, palatable rice, making it suitable for all ages. While there have been numerous studies on roasted peanuts (11), peanut butter (12), and peanut oil (13), quantitative analysis of volatile flavor components in peanut porridge remains scarce. However, research on the flavor components of peanut porridge remains limited, particularly with respect to the quantitative analysis of volatile compounds, which has not yet been systematically addressed. Conducting a comprehensive analysis of these components will help clarify the mechanisms by which different processing methods influence the flavor profile of peanut porridge, thereby providing a theoretical foundation for product standardization, industrial-scale production, and flavor quality optimization.

Electronic sensory analysis technology uses advanced instruments to simulate human sensory organs, with common techniques including computer vision, electronic nose, and electronic tongue. These technologies can capture extensive information describing the flavor profiles and taste components of products. Compared with traditional sensory analysis methods that rely on trained panels, electronic nose and electronic tongue systems offer rapid, objective, and reproducible detection of volatile flavor compounds by mimicking human olfactory and gustatory mechanisms, thereby reducing human error and enabling standardized evaluation of food flavor characteristics. Specifically, the electronic nose employs a sensor array to rapidly respond to volatile compounds, completing odor profile analysis within minutes. For example, in tea aroma assessment, the electronic nose not only analyzes aroma characteristics but also maps semantic information associated with variety and grade (14). Similarly, the electronic tongue simulates human taste perception and quantifies basic taste attributes such as sourness, sweetness, bitterness, saltiness, and umami. For instance, in differentiating edible fungi species, the electronic tongue successfully analyzed and identified umami-related compounds in 12 mushroom varieties, enabling accurate species discrimination (15).

During peanut processing, chemical reactions such as the Maillard reaction (16), protein denaturation (17), and lipid oxidation (18) may occur, leading to the generation of a wide range of volatile flavor compounds. Although quantitative analysis cannot directly prevent protein denaturation or lipid oxidation, monitoring changes in aroma compounds provides a sensitive indicator of the extent of these thermal reactions. Therefore, dynamic variations in aroma profiles can serve as critical references for assessing the intensity of thermal reactions and quality deterioration during processing, facilitating optimization of process parameters and stability control, which ultimately exert significant influence on the flavor quality of the final product. Currently, techniques such as gas chromatography–olfactometry (GC-O), gas chromatography–mass spectrometry (GC–MS), and GC-IMS are widely employed for the identification and quantification of volatile flavor compounds in food (19). Compared with conventional GC–MS, GC-IMS offers advantages such as simple operation, high sensitivity, and the ability to retain the original aroma of samples. It has been widely applied in studies related to shelf-life evaluation, volatile component analysis, geographical origin authentication, and quality assessment of various foods (20). Deng et al. (21), in a study combining GC-IMS and GC–MS for the volatile characterization of walnut oil extracted by aqueous enzymatic and refined processes, reported that GC-IMS exhibits higher sensitivity than GC–MS, enabling detection of small-molecule and low-concentration volatile compounds and thus expanding the detectable range of volatiles in samples. Consequently, GC-IMS has emerged as an advanced technique for detecting volatile flavor compounds in foods (19). This technology integrates the advantages of electronic sensory and chromatographic analysis: electronic sensory systems provide an overall flavor profile, while GC-IMS enables detailed identification of specific volatile compounds, thus allowing a comprehensive characterization of flavor attributes (22). The adoption of multi-technique analytical strategies not only enhances detection efficiency and accuracy but also offers distinct benefits such as shorter analysis time, elimination of sensory fatigue, rich information output, and strong fault tolerance, making it highly promising for flavor research and quality control in the food industry (23).

Given the advantages of GC-IMS—such as the absence of sample pretreatment, high sensitivity, and capability to detect low-abundance compounds—this study employed GC-IMS to investigate the volatile fingerprint characteristics of peanut porridges prepared under different processing conditions. PLS-DA was applied to establish a robust and predictive model, and characteristic volatile flavor compounds were screened based on VIP values. Combined with PCA radar charts, heatmaps, and other visualization tools, was performed using data from electronic sensory analysis and volatile compound profiling to differentiate samples prepared from various treatments. Particular emphasis was placed on comparing the major aroma differences between porridges made from aged and fresh peanuts. By quantitatively analyzing key volatile compounds, this study aims to provide a scientific basis for flavor optimization, process parameter adjustment, product development, and flavor consistency control in peanut porridge, thereby promoting the standardization and industrialization of this traditional product.

## 2 Materials and methods

### 2.1 Materials and reagents

Aged peanuts and fresh peanuts (variety: Xujiao No. 4), along with polished rice, were purchased from the agricultural market on Lianhu Road, Hanzhong City, Shaanxi Province, China. All samples were vacuum-packed and stored at 4 °C prior to use.

### 2.2 Instruments and equipment

The instruments used in this study included: SuperTongue electronic tongue (ISENSO Group, France), SuperNose electronic nose (ISENSO Group, France), DigiEye color measurement system (VeriVide Ltd., UK), MJ-BL10S11 high-speed blender (Midea Co., Shunde, China), ZB-3005A moisture analyzer (Zhengruitai Electronic Technology Co., Jiangsu, China), and FlavourSpec gas chromatography–ion mobility spectrometer (GC-IMS; Dortmund Co., Germany).

### 2.3 Experimental methods

#### 2.3.1 Peanut processing

For the aged peanut samples, appropriate amounts were cleaned to remove surface dust and impurities, soaked in water for 4 h, and then ground using a high-speed blender. The resulting slurry was mixed with a suitable amount of rice and boiled to prepare peanut porridge, with foam skimmed off during boiling.

For the fresh peanuts, the shells were removed, and defective or damaged kernels were discarded. The kernels were washed, blended into a slurry, mixed with rice, and boiled similarly. The detailed treatment groups were as follows:

##### 2.3.1.1 Aged peanut groups

LXF: 80 g aged peanut slurry + 16 g rice + 560 mL purified water; boiled on an induction cooker with continuous stirring. LYJZ: 80 g aged peanut slurry + 560 mL purified water; boiled with stirring. LT: 40 g soaked aged peanuts (after 4 h soak) + 600 mL purified water; boiled and stirred. LYJ: Raw aged peanut slurry.

##### 2.3.1.2 Fresh peanut groups

XXF: 80 g fresh peanut slurry + 16 g rice + 560 mL purified water; boiled with stirring. XYJZ: 80 g fresh peanut slurry + 560 mL purified water; boiled and stirred. XT: 40 g fresh peanuts + 600 mL purified water; boiled and stirred. XYJ: Raw fresh peanut slurry.

In this study, only fresh peanuts were subjected to peeling treatment, primarily because fresh peanuts have a higher moisture content and a thinner seed coat, making peeling easier. If not peeled, bitter compounds may be released during cooking, adversely affecting the final product’s flavor (24). In contrast, aged peanuts, after long-term storage, develop a harder and more tightly adhered seed coat, resulting in low peeling efficiency. Moreover, studies have shown that peeling has little effect on the final aroma composition of aged peanuts (25). Therefore, to better reflect practical processing scenarios while preserving the natural flavor evolution, aged peanuts were not peeled in this work.

#### 2.3.2 Electronic tongue and electronic nose analysis

The prepared peanut porridge samples and raw slurries of fresh and aged peanuts were diluted at a ratio of 1:10 (w/v) with purified water. The diluted samples were then centrifuged, and the supernatant was collected for analysis. For electronic tongue (E-tongue) testing, 25 mL of supernatant was transferred into a dedicated E-tongue beaker. For electronic nose (E-nose) analysis, 10 mL of supernatant was added to a standard E-nose reagent vial. In total, eight groups of samples were analyzed, each in triplicate. The taste and odor profiles were evaluated using the electronic tongue and nose, respectively. The sensor array used in the E-nose is listed in Table 1. The electronic nose used in this study is equipped with 14 types of sensors, covering a range of volatile organic compounds (VOCs) including alcohols, aldehydes, esters, ketones, alkanes, sulfur-containing compounds, and nitrogen-containing heterocycles. These compounds are generated during peanut porridge processing through lipid oxidation, Maillard reactions, protein degradation, and other processes. The use of this sensor array enables broad-spectrum detection of complex aroma components, enhancing both the comprehensiveness and sensitivity of the analysis.

**Table 1:** Electronic nose sensor table.

ID	Sensor ID	Target analytes
1	S1	Alkanes and smoke-related compounds, such as propane, natural gas, and smoke
2	S2	Alcohols, aldehydes, and short-chain alkanes, such as ethanol, smoke, isobutane, and formaldehyde
3	S3	Ozone (at low concentrations)
4	S4	Sulfides, such as hydrogen sulfide
5	S5	Nitrogen-containing compounds, such as nitrogen oxides
6	S6	Organic gases, ketones, alcohols, aldehydes, aromatic compounds, such as toluene, acetone, ethanol, hydrogen, and other organic vapors
7	S7	Ketones and alcohols, such as acetone, ethanol, propylene glycol, and organic solvents
8	S8	Short-chain alkanes, such as propane and liquefied petroleum gas
9	S9	Partial organic solvents, including alcohols, ethers, esters, ketones, aromatic hydrocarbons, aliphatic hydrocarbons, alicyclic hydrocarbons, and halogenated hydrocarbons
10	S10	Hydrogen-containing gases, such as hydrogen
11	S11	Allyl sulfides, such as methyl allyl trisulfide
12	S12	Short-chain alkanes, such as liquefied gas and methane
13	S13	Short-chain alkanes, such as methane, natural gas, and biogas
14	S14	Flammable gases, such as combustible gases and smoke

#### 2.3.3 GC-IMS analysis

Volatile compounds in the samples were analyzed using a FlavourSpec® gas chromatography–ion mobility spectrometry (GC-IMS) instrument (G. A. S. mbH, Dortmund, Germany) with an automated headspace sampling system. The headspace sampling conditions were as follows: incubation temperature, 40 °C; incubation time, 10 min; agitation speed, 500 rpm; injection volume, 1 mL; injection needle temperature, 85 °C; splitless mode.

The IMS conditions were: analysis time, 30 min; IMS temperature, 45 °C; drift gas, high-purity nitrogen (≥99.999%). The GC column flow rate was programmed as follows: initial flow rate of 2 mL/min for 2 min; ramped from 2 to 15 mL/min during 2–10 min; ramped from 15 to 100 mL/min during 10–25 min; held at 100 mL/min from 25 to 30 min.

The chromatographic separation was performed on an MXT-5 capillary column (15 m × 0.53 mm, 1 μm film thickness) at a constant column temperature of 60 °C. High-purity nitrogen (≥99.999%) was used as the carrier gas. Compound identification was carried out using VOCal software (G. A. S.) with integrated NIST and IMS databases. 2-Methyl-3-heptanone was used as an internal standard.

### 2.4 Data processing

Topographic plots and gallery plots were generated using the instrument software LAV, Reporter, and Gallery Plot. Qualitative analysis of volatile compounds was performed based on the GC × IMS Library Search using the integrated NIST and IMS databases. The relative contents of volatile compounds were calculated based on normalized peak volumes.

Statistical analysis was conducted using SPSS 22.0, and significant differences were evaluated using Duncan’s multiple range test at a significance level of *p* < 0.05. Graphical visualization was performed using Origin 2021. Partial least squares-discriminant analysis (PLS-DA) was performed using the SIMCA 14.1 software and the online Metware Cloud platform. Volatile markers were screened based on variable importance in projection (VIP) values greater than 1, and differences in volatile profiles among different treatments were further analyzed.

## 3 Results and analysis

### 3.1 Electronic tongue

PCA is a classical dimensionality reduction and pattern recognition method, particularly suitable for visualizing high-dimensional, multivariate flavor data and conducting preliminary clustering analysis. PCA intuitively displays differences and distribution trends among samples, laying the foundation for subsequent discriminant analysis and flavor compound screening (26). In contrast, other methods such as hierarchical cluster analysis (HCA) or discriminant analysis (DA) are typically employed for later-stage classification or supervised learning. Therefore, PCA is widely used in flavor profile identification (27).

**Figure 1:**
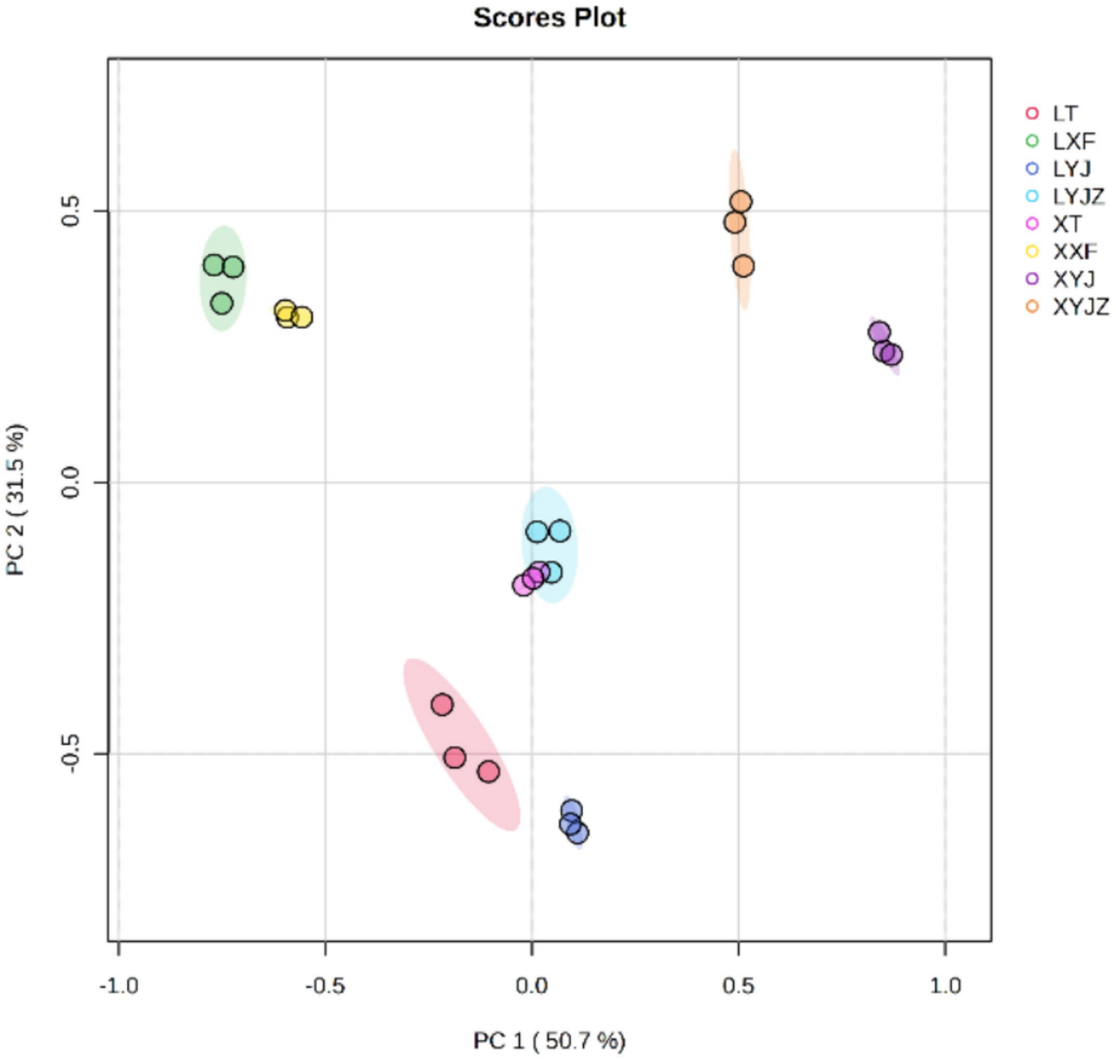
The PCA score plot of the electronic tongue.

The electronic tongue, which simulates human gustatory perception, was used to analyze the taste characteristics of the samples. This technique is fast and convenient and can serve as a substitute or supplement to traditional sensory evaluation. PCA was conducted on the data obtained by the electronic tongue. As shown in Figure 1, The contribution rates of the first and second principal components (PC1 and PC2) were 50.7 and 31.5%, respectively, with a cumulative contribution rate of 82.2%. This indicates significant differences among the sample groups, with no overlap between them, demonstrating that the electronic tongue technology can effectively distinguish among the different samples.

### 3.2 Electronic nose

**Figure 2:**
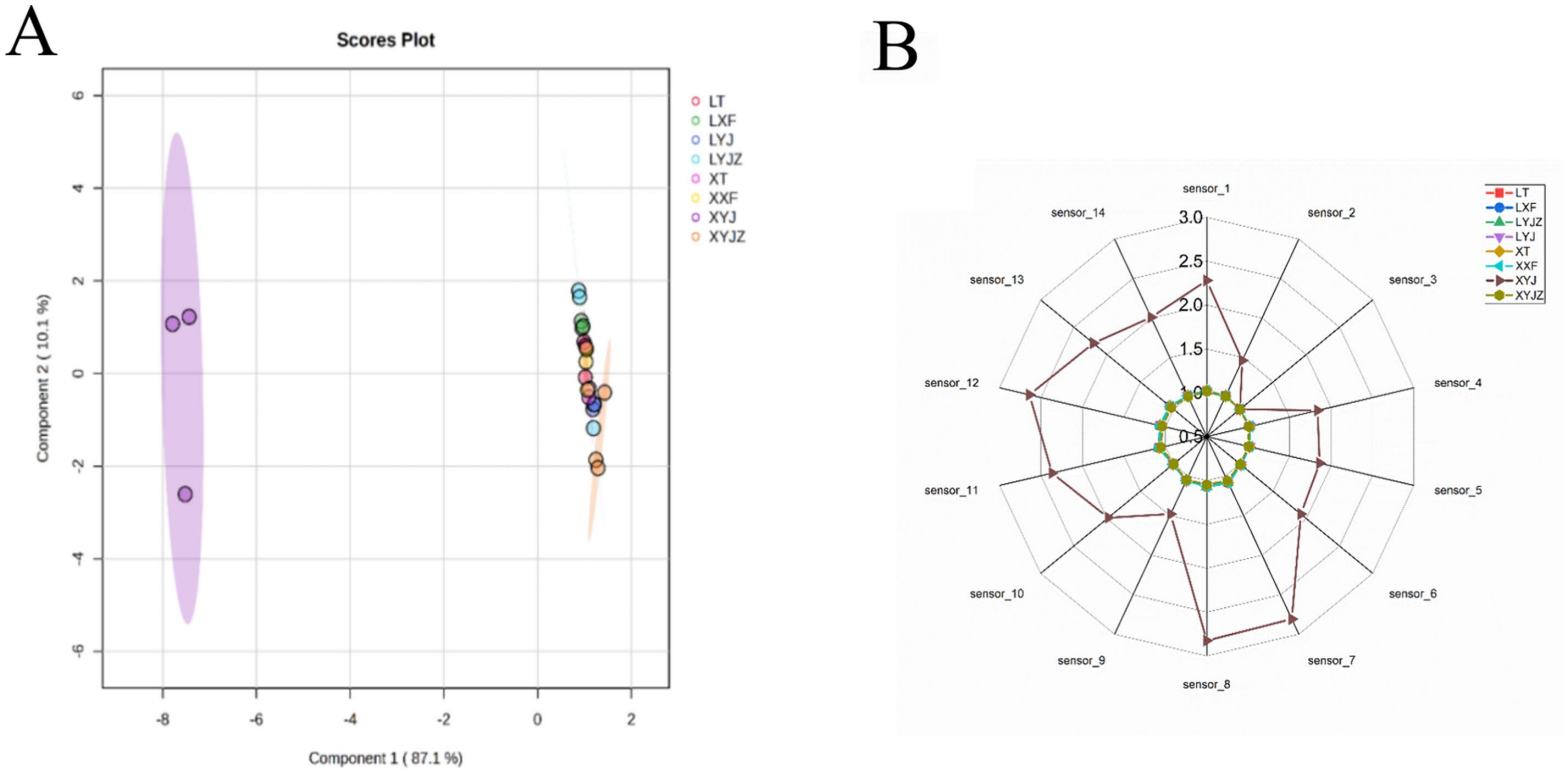
**(A)** PCA score plot of electronic nose; **(B)** Radar plot of electronic nose.

The odor profiles of the eight peanut samples were modeled using the edit function in the electronic nose software, which is typically used to edit or modify data, configurations, parameters, or settings for odor or gas sensor data analysis. PCA was then applied for further analysis, and the results are presented in Figure 2A. As shown in Figure 2A, the total variance explained by the first two principal components was 97.2%, with PC1 and PC2 contributing 87.1 and 10.1%, respectively. PC1 exhibited strong discriminatory power, while PC2 contributed less to group separation. This suggests that electronic nose analysis combined with PCA can effectively differentiate peanuts processed in different ways based on their volatile compounds. Notably, the fresh peanut slurry sample (XYJ) was located far from the other groups, indicating that the electronic nose could clearly identify the differences in volatile compounds among samples.

As shown in Figure 2B, the taste profile of the fresh peanut slurry differed markedly from the other samples. According to the corresponding electronic nose sensor responses, the fresh peanut slurry contained lower levels of ozone-like compounds, suggesting a lower degree of oxidation. In contrast, the levels of alkanes, ketones, and alcohols were relatively higher. These variations in volatile organic compounds (VOCs) may be attributed to lipid degradation and the Maillard reaction, which are enhanced under moist heat conditions (28). This indicates that steaming has a pronounced effect on the flavor development of fresh peanuts.

### 3.3 Analysis of volatile flavor compounds in Peanut porridge by GC-IMS

#### 3.3.1 3D topographic plots and comparative analysis of volatile compounds

**Figure 3:**
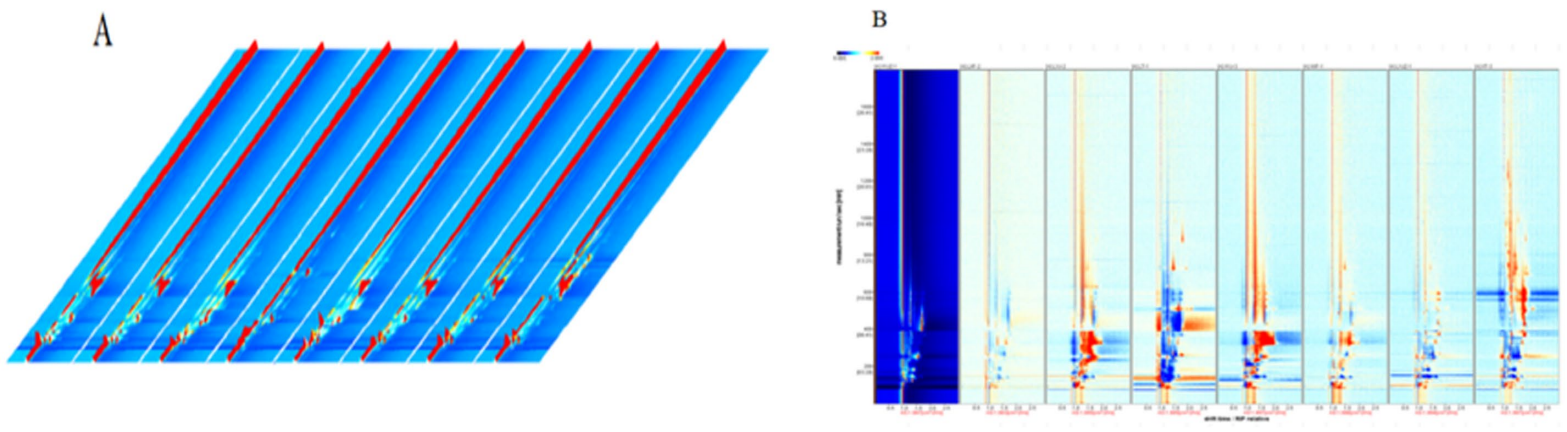
**(A)** Three-dimensional GC-IMS topographic plot of volatile compounds among samples; **(B)** Difference plot.

To facilitate intuitive observation and comparison, a three-dimensional (3D) topographic plot was employed to characterize the aroma compounds in peanut porridge. Figure 3A presents the 3D topographic plots of VOCs in eight differently treated groups. In the plot, the X-axis, Y-axis, and Z-axis represent the drift time of the identified ions, the retention time in gas chromatography, and the quantitative peak height, respectively. Each point in the plot represents a specific aroma compound, and the closer the color is to red, the higher the signal intensity. As shown in the figure, with sample XYJZ-1 as the control, VOCs in peanut porridge could be effectively detected using GC-IMS. This analysis lays the foundation for qualitative identification, revealing notable differences in signal intensities among aroma compounds in different samples, thereby enabling GC-IMS to better differentiate compounds between samples.

To more clearly visualize the differences in volatile aroma compounds among samples, a difference spectrum of peanut porridge was constructed using GC-IMS. Based on the GC-IMS detection results, a differential model plot was generated. After normalizing the retention time and signal intensity of the aroma compounds, each point in the plot represents a compound. A white background indicates that the compound concentration is similar to that in the control peanut porridge, while blue regions indicate lower concentrations and red regions higher concentrations (29). As shown in Figure 3B, each distinct point represents a different VOC, which facilitates visual analysis. Using sample XYJZ-1 as the reference, the VOC compositions of other samples showed generally consistent differences, which agrees with the results of the 3D topographic plots.

This study demonstrates that the GC-IMS characteristic spectra of volatile compounds in peanut porridge varied under different treatments. These differences may result from Maillard reactions occurring in peanut kernels (30), which produce nitrogen- and oxygen-containing heterocyclic compounds such as pyrazines, pyrroles, pyridines, and furanones. These VOCs contribute significantly to the characteristic aroma of peanut porridge (31).

#### 3.3.2 Quantitative analysis of volatile flavor compounds by GC-IMS

Based on the retention index data from the NIST database integrated within the FlavourSpec® software and the drift time information from the G. A. S. IMS library, a total of 47 VOCs were identified across samples processed for different cooking durations (see Table 2). Among them, 10 were alcohols, 10 esters, 6 ketones, 5 acids, 2 alkenes, 6 aldehydes, and 8 other types of compounds Based on the identified volatile compounds, it can be inferred that alcohols, aldehydes, ketones, alkenes, acids, and esters are the major contributors to the aroma of peanut porridge. The 47 volatile compounds listed in the table were generally consistent with those reported by Sumin Ma et al. (32) in their review of production techniques, flavor compounds, formation mechanisms, and influencing factors of roasted peanut oil. Among these, furan compounds are key aroma contributors formed during the thermal processing of nuts and oilseeds; aldehydes, as lipid degradation products, have a significant impact on flavor and impart umami notes (11); acids primarily originate from the hydrolysis of peanut fatty acids, and their content and types are influenced by storage duration and conditions (33); hydrocarbons are generated through the cleavage of lipid peroxides (34), providing aromatic and slightly pungent notes; in addition, Maillard reactions occurring in peanuts may produce ketones, which are characterized by strong volatility and a rich nutty or roasted aroma (35). Alcohols and esters, as natural constituents of peanuts, were detected across all treatments, contributing to the unique aroma profile of peanut porridge. The characteristic aroma of peanuts is not dominated by a single compound or a few compounds but rather reflects the synergistic effect of multiple components (6). Based on the GC-IMS fingerprints and Table 2, the raw slurry of both fresh and aged peanuts exhibited a more diverse composition of flavor compounds compared to other groups. After steaming, although the concentration of certain volatile compounds decreased, the levels of key aroma-contributing substances increased, resulting in a more pronounced aroma in the final peanut porridge. Future work could focus on targeted control of processing parameters (e.g., temperature and time) to optimize the generation and retention of flavor compounds, thereby providing a scientific basis for improving the quality of peanut-based products.

**Table 2:** Final volatile components identified in the porridge.

ID	Category	Compound name	RI	Rt [sec]	Dt [a.u.]	Relative abundance/%							
						XYJZ	LXF	LYJ	LT	XYJ	XXF	LYJZ	XT
1	Alcohols	1-Heptanol	957	427.504	1.74837	0.89 ± 0.03 cd	0.94 ± 0.06 cd	0.49 ± 0.16de	0.19 ± 0.04e	1.16 ± 0.38bc	1.14 ± 0.26bc	1.69 ± 0.76b	3.02 ± 0.21a
2		Pentan-1-ol	774.4	234.151	1.25284	1.4 ± 0.07bc	1.67 ± 0.15b	0.11 ± 0.12e	4.4 ± 1.02a	1.06 ± 0.41bcd	1.5 ± 0.21bc	0.77 ± 0.25cde	0.35 ± 0.10de
3		Ethanol	500.3	96.176	1.13233	0.36 ± 0.11c	0.34 ± 0.08c	6.08 ± 0.39b	6.02 ± 0.15b	14.98 ± 2.51a	1.31 ± 1.52c	0.67 ± 0.52c	1.57 ± 0.76c
4		1-Octene-3-ol	1007.3	509.945	1.74469	0.08 ± 0.06c	0.12 ± 0.01c	0.8 ± 0.38b	2.11 ± 0.23a	1.13 ± 0.37b	0.21 ± 0.12c	0.07 ± 0.04c	0.03 ± 0.02c
5		n-Hexanol	893	336.177	1.99391	0.13 ± 0.03 cd	0.14 ± 0.02 cd	1.07 ± 0.17b	0.38 ± 0.08c	2.07 ± 0.3a	0.39 ± 0.23c	0.09 ± 0.02d	0.08 ± 0.02d
6		2-Methyl-1-propanol	626.3	149.064	1.37122	2.51 ± 0.12a	2.16 ± 0.24ab	1.42 ± 0.12ab	0.18 ± 0.04c	0.95 ± 0.58c	1.27 ± 0.69bc	1.36 ± 1.16abc	1.12 ± 1bc
7		3-Octen-1-ol, (Z)-	1041.8	592.986	1.74707	0.32 ± 0.04 cd	0.22 ± 0.04 cd	0.18 ± 0.02d	0.23 ± 0.04 cd	0.43 ± 0.12bc	0.45 ± 0.08bc	0.6 ± 0.33b	1.99 ± 0.07a
8		2-Ethyl-1-hexanol	1041.7	592.717	1.79216	0.4 ± 0.06c	0.27 ± 0.06c	0.25 ± 0.04c	0.21 ± 0.03c	0.47 ± 0.2c	0.67 ± 0.09bc	0.96 ± 0.56b	6.27 ± 0.41a
9		2-Methylbutan-1-ol	780	237.976	1.47799	1.32 ± 0.03c	1.29 ± 0.24c	2.31 ± 0.14b	1.52 ± 0.18bc	3.17 ± 1.12a	1.5 ± 0.27bc	1.53 ± 0.51bc	1.25 ± 0.17a
10		(E)-2-hexen-1-ol	851.9	299.913	1.51073	0.37 ± 0.04b	0.20 ± 0.03bc	2.7 ± 0.27a	0.27 ± 0.06bc	2.47 ± 0.26a	0.3 ± 0.06bc	0.13 ± 0.03bc	0.07 ± 0.00c
11	Aldehydes	Heptanal	919.6	374.144	1.32216	1.78 ± 0b	1.74 ± 0.16b	0.34 ± 0.13d	0.15 ± 0.07d	1.09 ± 0.07c	1.41 ± 0.56bc	3.16 ± 0.62a	2.66 ± 0.23a
12		Hexanal	826.6	277.792	1.26268	0.06 ± 0.02c	0.06 ± 0.03c	1.2 ± 0.02a	0.11 ± 0.05c	0.36 ± 0.23b	0.21 ± 0.23bc	0.08 ± 0.04c	0.19 ± 0.1bc
13		Pentanal	700.8	183.577	1.19529	4.39 ± 0.1b	4.61 ± 0.19b	0.01 ± 0.03d	11.9 ± 1.29a	0.01 ± 0.02d	3.68 ± 0.39bc	2.91 ± 0.91c	0.86 ± 0.11d
14		Butanal	582	130.451	1.13205	13.04 ± 0.68a	13.23 ± 0.25a	11.94 ± 0.72ab	0.21 ± 0.07d	3.55 ± 0.39c	12.14 ± 2.16ab	13.42 ± 1.71a	10.67 ± 1.24b
15		3-Methylbutanal	650.1	159.031	1.19826	0.45 ± 0.07d	0.66 ± 0.06d	7.52 ± 0.44a	1.4 ± 0.04 cd	5.68 ± 1.26b	1.14 ± 0.7 cd	0.88 ± 0.49d	2.34 ± 1.04c
16		(E)-2-pentenal	763.3	226.468	1.09155	0.25 ± 0.04b	0.18 ± 0.04b	0.48 ± 0.05b	0.64 ± 0.14b	2.81 ± 0.33a	0.36 ± 0.17b	0.48 ± 0.58b	0.17 ± 0.06b
17	Alkenes	?alpha?-Phellandrene	991.1	476.167	1.69309	1.08 ± 0.85c	0.39 ± 0.52c	0.19 ± 0.06c	28.38 ± 1.91a	0.4 ± 0.65c	0.25 ± 0.1c	0.38 ± 0.18c	4.77 ± 1.28b
18		Limonene	1,042	593.543	1.291	1.89 ± 0.16c	1.84 ± 0.07c	1.02 ± 0.07e	0.22 ± 0.06f	1.46 ± 0.26d	2.47 ± 0.17b	2.83 ± 0.19a	0.86 ± 0.35e
19	Esters	Acetic acid butyl ester	808.5	262.059	1.61277	0.44 ± 0.09ab	0.25 ± 0.05ab	0.01 ± 0.02b	0.13 ± 0.02ab	0.01 ± 0.01b	0.15 ± 0.07ab	1.02 ± 1.2a	0.52 ± 0.57ab
20		Acetic acid ethyl ester	608	141.356	1.07786	4.18 ± 0.28a	4.01 ± 0.85a	3.97 ± 0.62a	0.66 ± 0.02b	3.42 ± 0.43a	3.1 ± 1.27a	3.84 ± 2.39a	3.12 ± 1.51a
21		Amyl acetate	915.3	367.996	1.79546	1.17 ± 0.07 cd	1.06 ± 0.12 cd	0.47 ± 0.02d	0.24 ± 0.04d	0.94 ± 0.62 cd	1.77 ± 0.41bc	2.65 ± 1.21b	7.19 ± 0.36a
22		Butanoic acid methyl ester	737.9	209.034	1.1499	0.78 ± 0.05ab	0.58 ± 0.07bc	1.03 ± 0.07a	0.14 ± 0.01d	0.52 ± 0.44bc	0.4 ± 0.19 cd	0.55 ± 0.09bc	0.53 ± 0.03bc
23		Ethyl hexanoate	1001.8	496.573	1.33982	0.46 ± 0.01de	0.53 ± 0.02 cd	0.27 ± 0.01e	0.44 ± 0.11de	0.55 ± 0.21 cd	0.72 ± 0.09c	0.98 ± 0.28b	1.5 ± 0.08a
24		Ethyl propanoate	667.7	166.395	1.15137	1.15 ± 0.02ab	1.13 ± 0.15ab	0.73 ± 0.12bc	1.23 ± 0.07a	0.69 ± 0.1c	0.69 ± 0.2c	1.43 ± 0.58a	0.55 ± 0.11c
25		Methyl 2-methylbutanoate	786	242.386	1.18283	0.73 ± 0.03c	0.89 ± 0.05c	0.09 ± 0.01d	0.83 ± 0.16c	0.15 ± 0.04d	0.71 ± 0.14c	1.63 ± 0.27b	2.07 ± 0.21a
26		ethyl heptanoate	1088.5	705.42	1.40694	0.16 ± 0.04b	0.14 ± 0.01b	0.15 ± 0.03b	0.29 ± 0.09b	0.15 ± 0.01b	0.2 ± 0.02b	0.17 ± 0.04b	1.43 ± 0.57a
27		Acetic acid, 2-methylpropyl ester	773.2	233.288	1.60385	0.07 ± 0.02a	0.09 ± 0.01a	0.4 ± 0.03a	0.25 ± 0.08a	0.19 ± 0.02a	0.07 ± 0.02a	0.57 ± 0.91a	0.07 ± 0.01a
28		Butyl butanoate	1003.4	500.549	1.82189	0.13 ± 0.04c	0.11 ± 0.04c	0.12 ± 0.03c	0.57 ± 0.02b	0.15 ± 0.02c	0.18 ± 0.01c	0.23 ± 0.09c	2.2 ± 0.53a
29	Ketones	2-Hexanone	800.5	255.085	1.504	0.09 ± 0.04b	0.08 ± 0b	0.3 ± 0.16a	0.17 ± 0.04ab	0.03 ± 0.03b	0.06 ± 0.04b	0.2 ± 0.25ab	0.05 ± 0.02b
30		pentan-2-one	699.4	182.559	1.38172	2.24 ± 0.09a	2.22 ± 0.25a	1.17 ± 0.1b	0.62 ± 0.05c	0.5 ± 0.14 cd	0.79 ± 0.39c	0.8 ± 0.15c	0.2 ± 0.11d
31		2-Heptanone	884.1	327.924	1.63525	0.67 ± 0.05e	1.45 ± 0.3 cde	3.45 ± 0.15ab	4.25 ± 0.5a	2.2 ± 0.36 cd	2.49 ± 1.42bc	0.58 ± 0.47e	1.27 ± 0.15de
32		Furaneol	1107.6	751.387	1.19641	0.13 ± 0.01b	0.11 ± 0.01b	0.15 ± 0.02b	0.17 ± 0.02b	0.15 ± 0.05b	0.16 ± 0.03b	0.15 ± 0.07b	1.93 ± 0.69a
33		5-Methyl-3-heptanone	944.8	410.046	1.68568	21.38 ± 0.85abc	23.62 ± 1.19ab	24.17 ± 0.74a	3.75 ± 5.35e	19.7 ± 0.46bc	19.01 ± 1.12c	21.12 ± 1.66abc	12.11 ± 1.85e
34		1-Octen-3-one	996.3	483.57	1.27925	4.62 ± 0.16ab	4.54 ± 0.11ab	2.41 ± 0.06c	0.34 ± 0.28d	2.33 ± 0.51c	4.03 ± 0.59b	4.75 ± 0.37ab	5.09 ± 0.69a
35	Acids	2-Methylbutanoic acid	822.4	274.204	1.46881	0.08 ± 0.03a	0.06 ± 0.02a	0.08 ± 0.04a	0.52 ± 0.39a	0.09 ± 0.08a	0.09 ± 0.04a	0.47 ± 0.65a	0.29 ± 0.19a
36		Acetic acid	635.9	153.052	1.05458	0.7 ± 0.14a	0.58 ± 0.29ab	0.08 ± 0.08b	0.25 ± 0.11ab	0.38 ± 0.54ab	0.48 ± 0.24ab	0.29 ± 0.04ab	0.59 ± 0.46ab
37		Butanoic acid	795.1	250.359	1.40176	1.44 ± 0.09b	1.57 ± 0.03b	1.02 ± 0.08c	1.99 ± 0.22a	0.68 ± 0.19d	1.16 ± 0.04c	1.13 ± 0.13c	0.12 ± 0.02e
38		Formic acid	525.8	106.883	1.0423	5.47 ± 0.35ab	2.82 ± 0.37bc	1.00 ± 0.22c	3.06 ± 1.24bc	2.08 ± 1.57bc	8.55 ± 4.53a	2.25 ± 0.57bc	2.8 ± 0.67bc
39		Propanoic acid	681.6	172.232	1.08962	1.03 ± 0.05ab	1.2 ± 0.3a	0.33 ± 0.08d	0.41 ± 0.08d	0.39 ± 0.12d	0.45 ± 0.28 cd	0.78 ± 0.18bc	0.49 ± 0.23 cd
40	Others	2-Acetylfuran	916.2	369.283	1.45211	0.65 ± 0.12b	0.77 ± 0.08a	0.35 ± 0.04c	0.18 ± 0.03d	0.34 ± 0.03c	0.37 ± 0.05c	0.51 ± 0.06c	0.28 ± 0.02 cd
41		Heptane, 2,2,4,6,6-pentamethyl-	1007.5	510.374	1.40348	0.37 ± 0.07c	0.56 ± 0.06c	0.68 ± 0.13bc	2.82 ± 0.72a	0.37 ± 0.1c	1.15 ± 0.2b	0.5 ± 0.04c	0.49 ± 0.03c
42		Hexamethylcyclotrisiloxane	802.4	256.757	1.45092	0.05 ± 0.01b	0.07 ± 0.02b	0.06 ± 0.01b	13.47 ± 1.63a	0.55 ± 0.19b	0.07 ± 0.04b	0.24 ± 0.28b	0.77 ± 0.46b
43		Octamethyltrisiloxane	852.2	300.14	1.55551	0.03 ± 0.01b	0.05 ± 0.01b	0.07 ± 0.02b	0.17 ± 0.04b	0.34 ± 0.26a	0.08 ± 0.06b	0.07 ± 0.01b	0.07 ± 0.02b
44		2-pentyl furan	997.7	486.738	1.25215	0.94 ± 0.22 cd	1 ± 0.23 cd	0.93 ± 0.3 cd	2.47 ± 0.16b	1.04 ± 0.27 cd	4.82 ± 1.66a	1.92 ± 0.81bc	0.17 ± 0.04d
45		Tert-butylmethylether	574	127.121	1.35573	19.12 ± 0.34ab	19.59 ± 0.62a	15.67 ± 0.35 cd	0.29 ± 0.07e	17.4 ± 0.77abc	16.64 ± 1.21bcd	18.37 ± 2.95ab	14.87 ± 1.80d
46		Hexanenitrile	891.3	334.235	1.56958	0.21 ± 0.01d	0.3 ± 0.06d	1.02 ± 0.05a	0.56 ± 0.05b	0.34 ± 0.15 cd	0.47 ± 0.18bc	0.23 ± 0.1d	0.29 ± 0.02d
47		Acetoin	746.9	215.226	1.03891	0.81 ± 0.09bcd	0.58 ± 0.04d	1.69 ± 0.10a	1.22 ± 0.49b	1.07 ± 0.41bc	0.73 ± 0.13 cd	0.58 ± 0.06d	0.66 ± 0.19 cd

#### 3.3.3 Gallery plot fingerprint of volatile compounds in Peanut porridge

**Figure 4:**
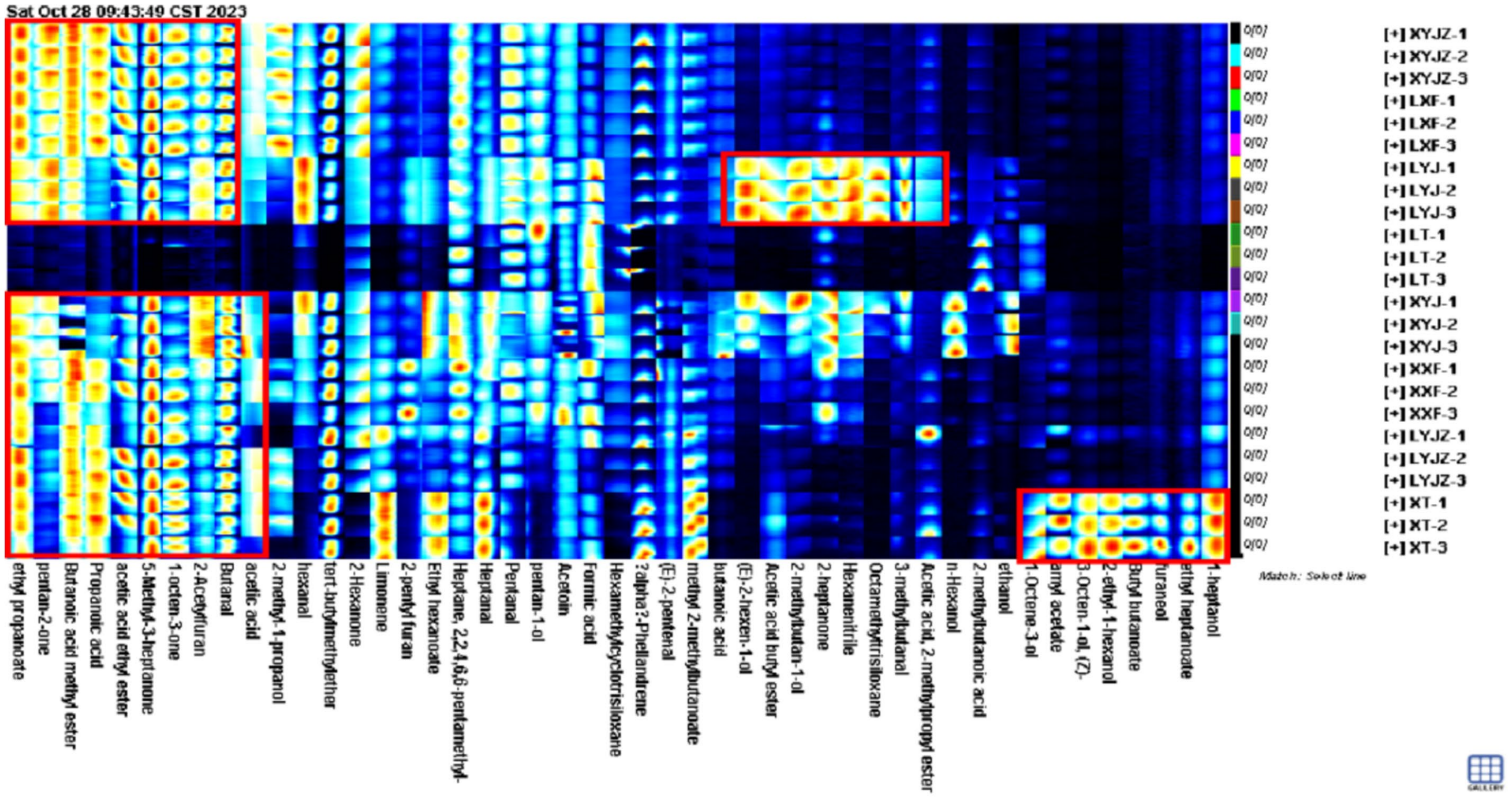
Relative content of volatile components.

To clearly illustrate the differences in volatile organic compounds (VOCs) among peanuts under different treatments, the peak volumes of various compounds in the GC-IMS fingerprints were normalized to obtain the relative contents of volatile components in peanut porridge. As shown in Figure 4, the volatile components were broadly classified into seven categories: aldehydes, alcohols, ketones, esters, alkenes, ethers, and others. Aldehydes accounted for approximately 14–22%, alcohols 8–29%, ketones 11–34%, esters 7–19%, ethers 15–20%, alkenes 1–30%, and others 16–21%. Additionally, the contents of these compounds varied with processing time. For example, in the LT group, alkenes and others reached 30 and 17%, respectively, whereas in other groups they remained between 1 and 6% and about 1%. The variation trends of esters were similar to those of alkanes. Acid compounds exhibited relatively stable contents with minor fluctuations. Ketones decreased significantly to 11% in the LT group, whereas in other groups their levels fluctuated between 20 and 34%. In the XYJ group, aldehydes were the lowest (14%), showing a decrease followed by an increase across treatments, while acids reached the highest level (29%) with an opposite trend. Other compounds remained relatively stable without major fluctuations.

**Figure 5:**
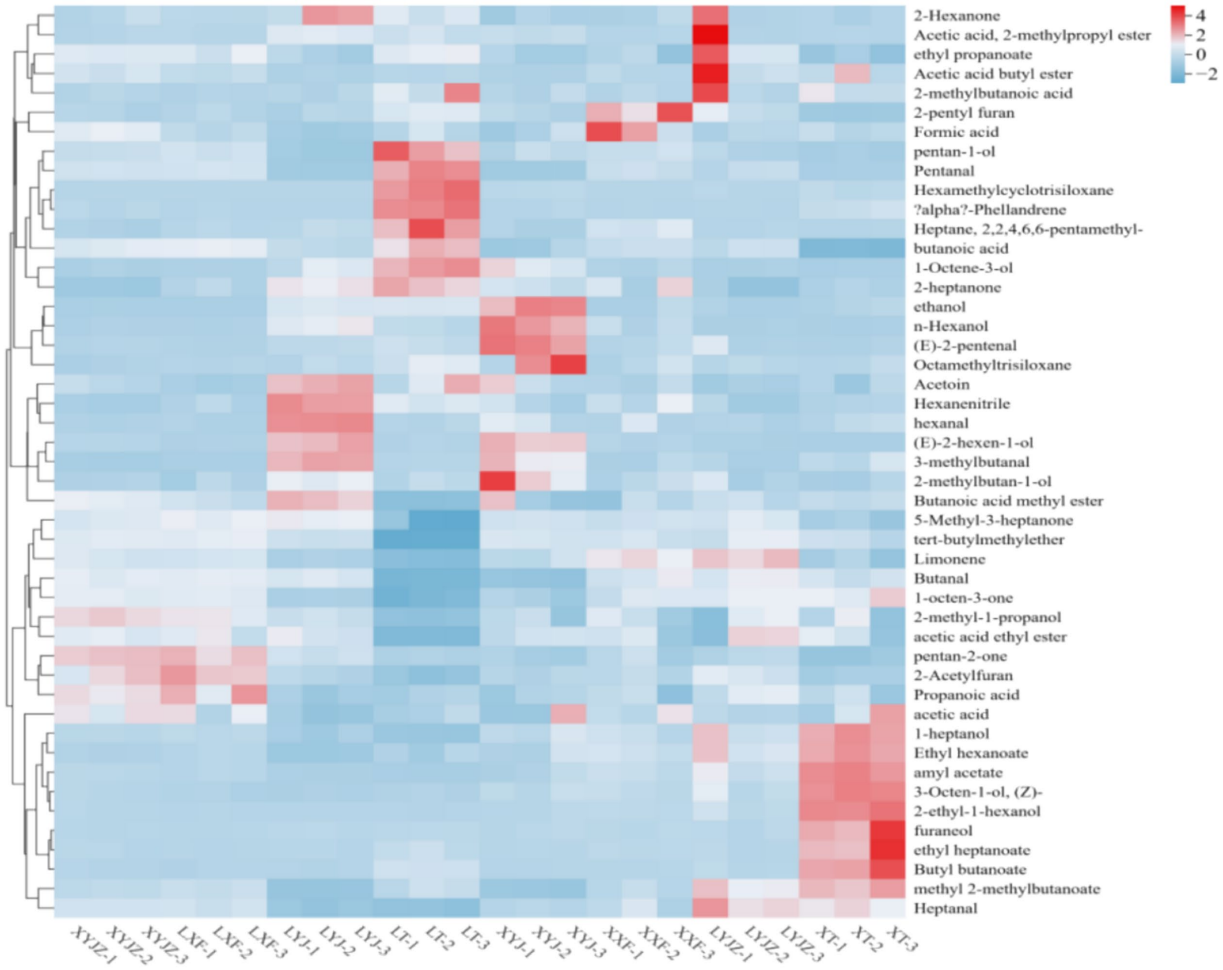
Heat map of volatile component clustering.

As shown in Figure 5, the clustered heatmap, with a color gradient from blue to red, accurately reflects the variation of each volatile compound from low to high levels, providing a clear visualization of differences among groups. Specifically, 1-pentanol, pentanal, hexamethylcyclotrisiloxane, p-mentha-1,5-diene, 2,2,4,6,6-pentamethylheptane, butanoic acid, and 1-octen-3-ol exhibited the highest intensities in the LT group, indicating significantly higher contents compared with other treatments. In contrast, 1-heptanol, ethyl hexanoate, amyl acetate, cis-3-octen-1-ol, 2-ethylhexan-1-ol, furfuryl alcohol, ethyl heptanoate, butyl butanoate, and methyl 2-methylbutanoate were dominant in the XT group. In the LYJ group, acetoin, hexanenitrile, hexanal, 2-hexen-1-ol, 3-methylbutanal, and methyl butanoate were most abundant, whereas the XYJ group showed higher levels of ethanol, 1-hexanol, trans-2-pentenal, and octamethyltrisiloxane. The LYJZ group exhibited high concentrations of heptanal, methyl 2-methylbutanoate, 2-hexanone, isobutyl acetate, ethyl propanoate, butyl acetate, and 2-methylbutanoic acid. These results indicate that clustered heatmaps effectively visualize VOC differences, and the distinct VOC profiles of porridge from different raw materials contribute to unique flavor signatures, providing valuable insights into the flavor formation mechanisms of peanut porridge.

**Figure 6:**
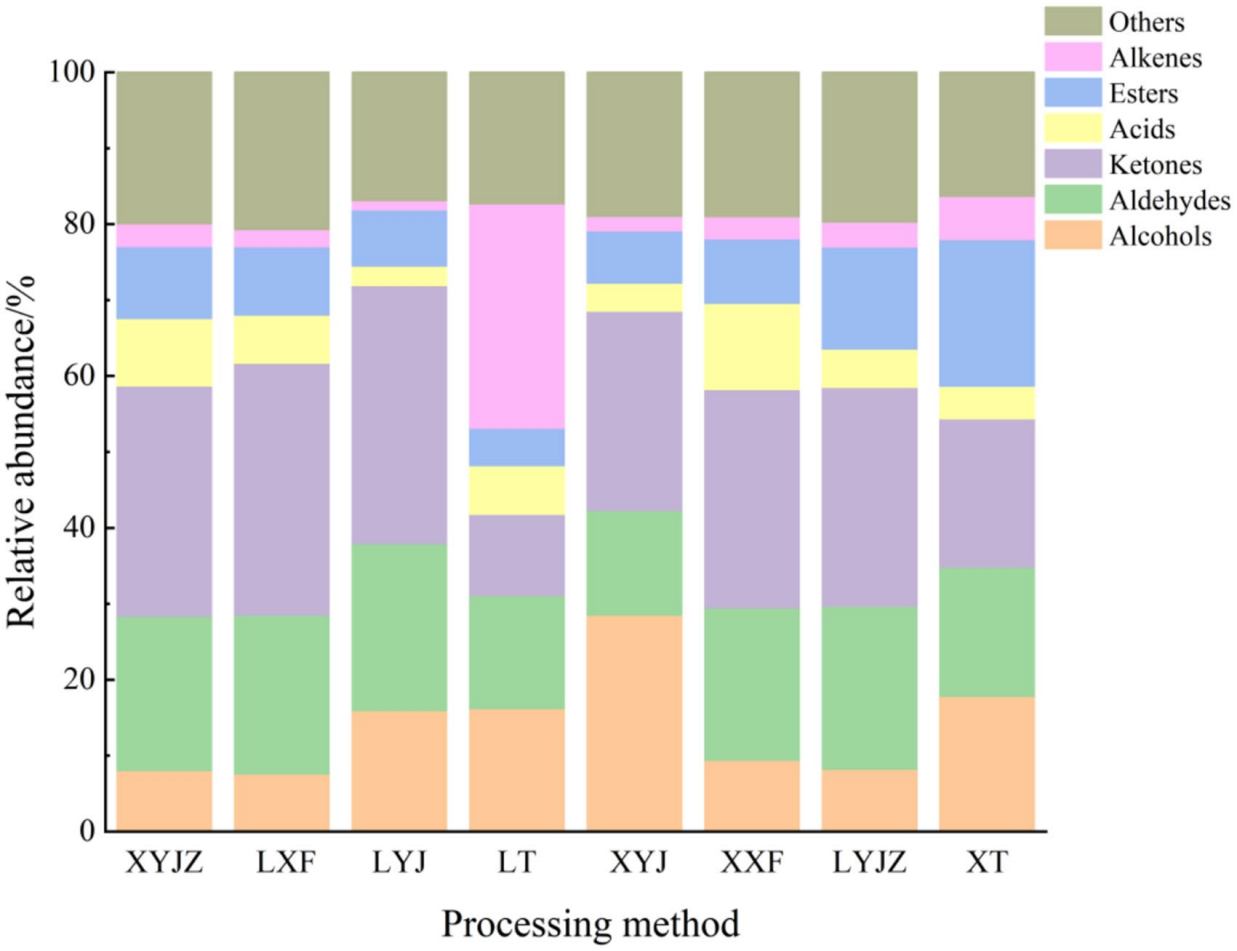
Gallery fingerprint map of the volatile components of peanut porridge.

Overall, Figures 4–6 reveal that sample XT was characterized by compounds such as 1-heptanol, ethyl heptanoate, furaneol, butyl butanoate, 2-ethyl-n-hexanol, 3-octen-1-ol, amyl acetate, and 1-octen-3-ol, with flavor dominated by the synergistic effects of esters (fruity, sweet) and alcohols (grassy, mushroom-like), likely derived from lipid oxidation or thermal degradation of ester precursors in peanuts. Samples LYJ and XYJ shared eight key compounds, including isobutyl acetate (fruity), 3-methylbutanal (malty), octamethyltrisiloxane (siloxane), and 2-heptanone (cheesy). Among them, LYJ exhibited higher intensities of butyl acetate (fruity) and 3-methylbutanal, resulting in stronger flavor intensity, while XYJ was distinguished by ethanol (alcoholic) and n-hexanol (grassy), conferring a fresher aroma profile. Sample LT was mainly characterized by acetoin (buttery) and aldehydes (pentanal, heptanal), combined with hexanenitrile (nutty bitterness) and 2,2,4,6,6-pentamethylheptane (alkane), forming a flavor dominated by buttery, grassy, and fatty notes, with acetoin likely originating from Maillard reactions. In XYJZ and LXF samples, esters such as ethyl propanoate (pineapple-like), 2,3-pentanedione (creamy), and methyl butanoate (apple-like) predominated, likely produced through esterification, contributing to intense fruity and dairy aromas.

In general, fresh peanuts were dominated by hexanal, hydrocarbons, and low-molecular-weight alcohols, presenting a relatively simple aroma profile. Previous studies by Brown (30) and Koji (36) also identified hexanal as a major aroma compound in fresh peanuts. Fresh peanut porridge mainly contained aldehydes, hydrocarbons, and alcohols, with hexanal as a key aroma contributor. In contrast, aged peanuts exhibited reduced ester levels but significantly higher aldehydes (e.g., hexanal) and alcohols (e.g., pentan-1-ol), resulting in stronger yet more diffusive flavor, likely associated with lipid oxidation and enzymatic reactions during long-term storage. Aged peanut porridge was dominated by ketones, possibly formed through Maillard reactions and accumulation of lipid oxidation products, imparting roasted and nutty aromas. Mechanical grinding of aged peanuts enhanced the release of esters and alcohols, although the overall content of volatile compounds decreased, possibly due to thermal degradation or volatilization of heat-sensitive substances (37).

### 3.4 PLS-DA and model validation

PLS-DA score scatter plots were used to classify the VOCs in peanut porridge samples under different treatment conditions. By reducing the dimensionality of the data, it becomes possible to identify and predict complex patterns. A corresponding model was constructed by randomly changing the order of the classification variables.

**Figure 7:**
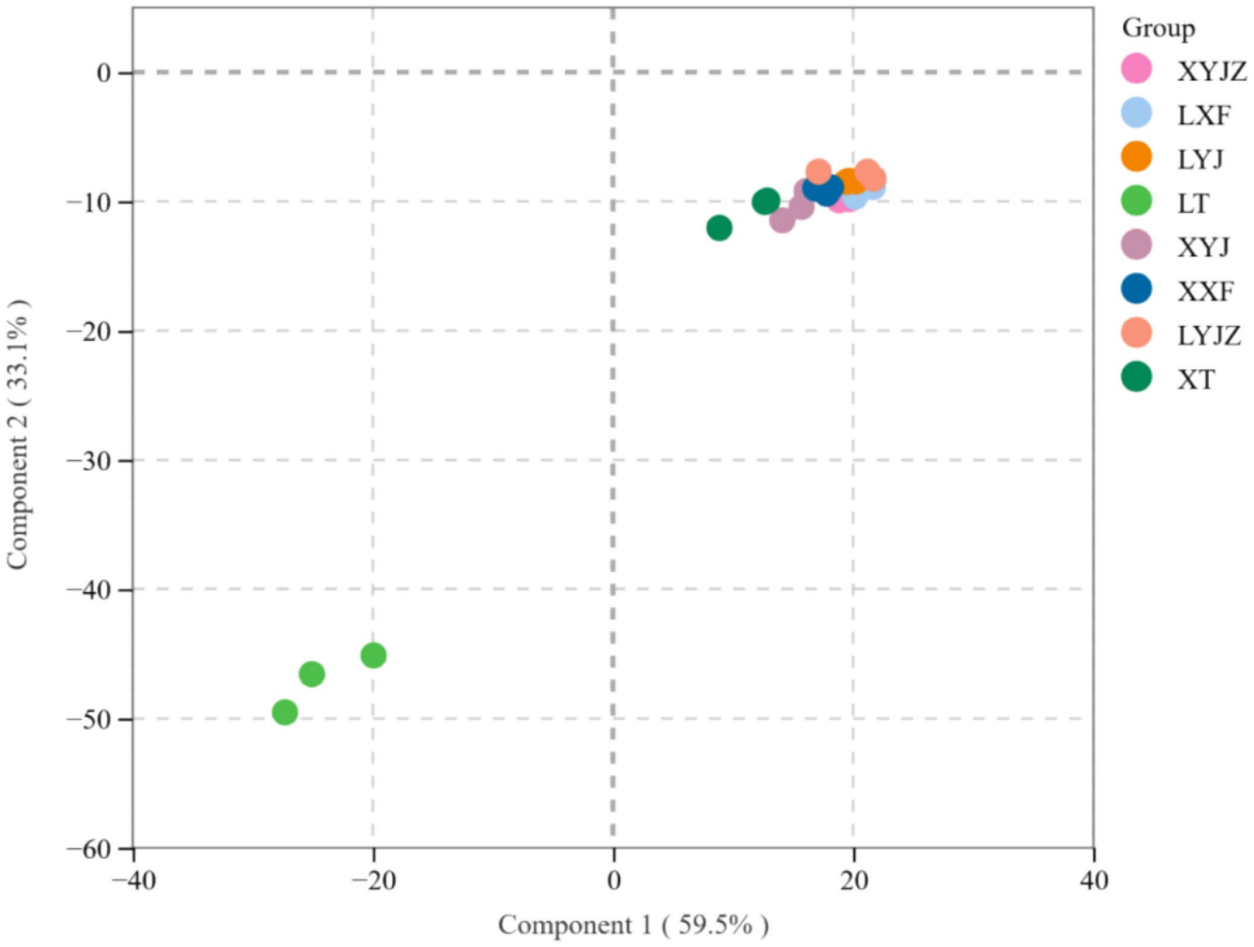
PLS-DA score plots.

As shown in Figure 7, the PLS-DA model yielded R^2^X = 0.83, R^2^Y = 0.948, and Q^2^ = 0.863. When both R^2^ and Q^2^ values fall within the range of 0.5 to 1.0, it indicates that the model has good generalization ability and explanatory power. The cumulative Q^2^ (Q^2^ cum = 0.863) further suggests that the model exhibits strong predictive capability. Samples (n = 200) were rearranged sequentially, and statistical test values were recalculated to create an empirical distribution. Based on this, the model was constructed. Q^2^ represents cumulative cross-validation, with its value being directly proportional to the model’s predictive power. R^2^ indicates the cumulative variance, reflecting the amount of original data used to build the PLS-DA discriminant model, with higher values representing greater explanatory power. When fitting the PLS-DA model, R^2^Y = 0.948 and Q^2^ = 0.863, indicating a good model fit.

**Figure 8:**
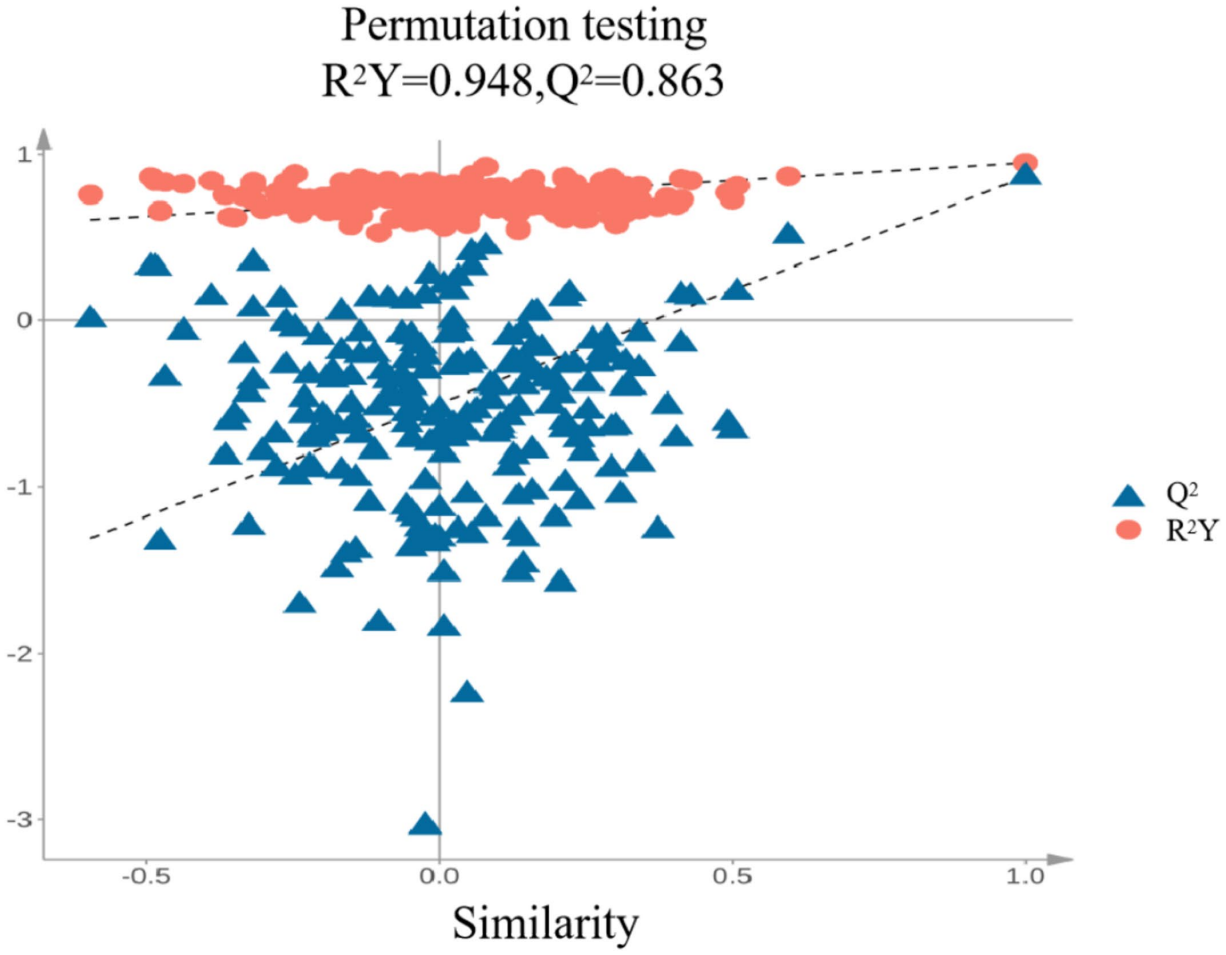
Displacement retention.

To further validate whether the model was overfitting, the categories of some samples were subject to 200 permutation tests. As shown in the right panel of Figure 8, the slopes of the R^2^ and Q^2^ regression lines are relatively steep. The R^2^ and Q^2^ values of the randomized experimental data (on the left) are lower than those of the original data (on the far right). Additionally, since the intercept of the Q^2^ regression line is negative, it can be concluded that there is no overfitting in the PLS-DA discriminant model. Therefore, the model is reliable for classifying and identifying the volatile components in peanut porridge under different treatments.

**Figure 9:**
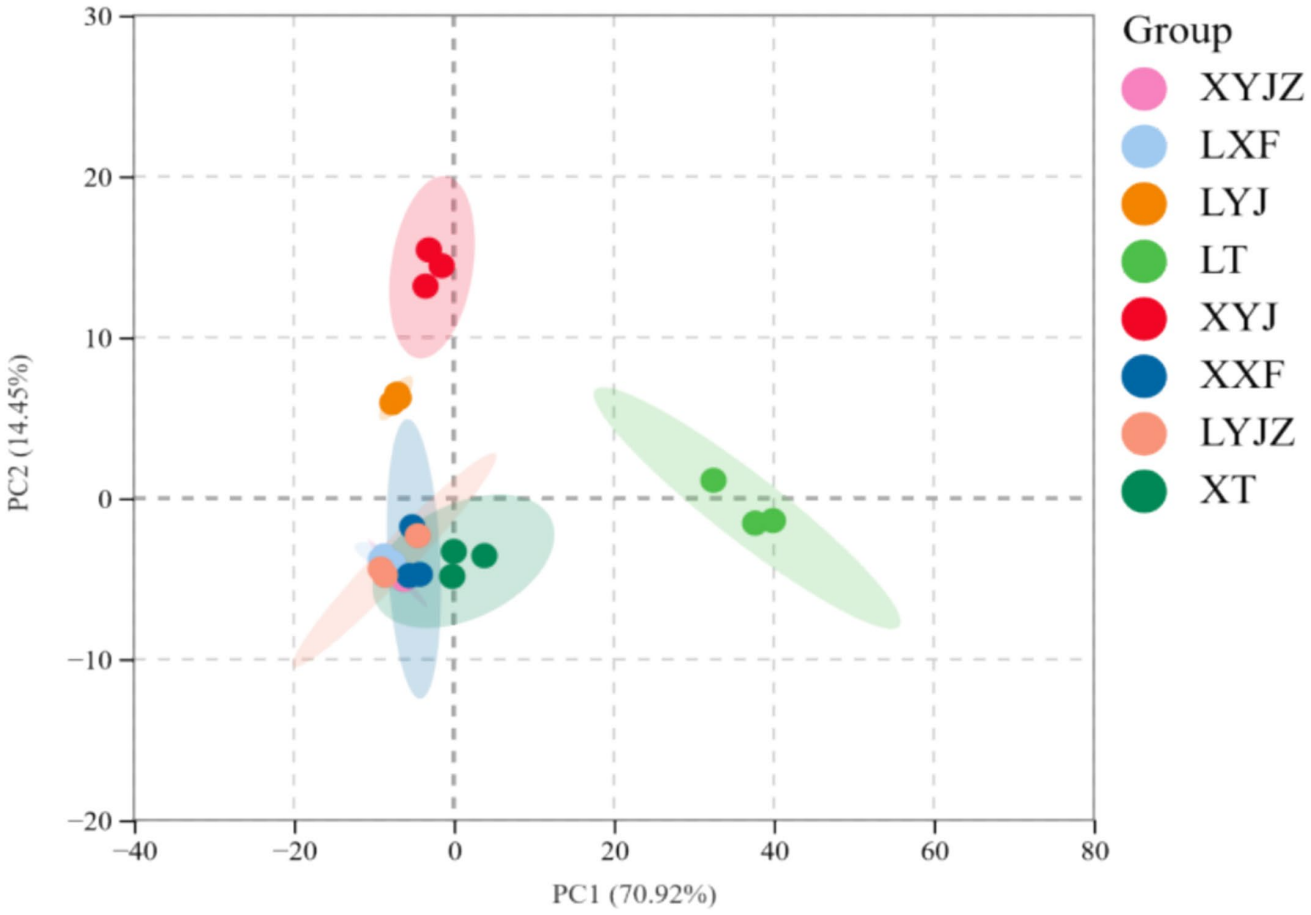
PCA score plots for all VOCs.

In Figure 9, PCA shows that PC1 accounts for 70.92%, and PC2 accounts for 14.45%, with a total contribution of 85.37%, exceeding 50%, which indicates a good separation effect. This suggests that the fresh peanut slurry significantly differs from the other seven treatment groups, while the fresh peanut soup and cooked old peanut slurry share similar chemical compositions. This result aligns with the electronic nose data.

**Figure 10:**
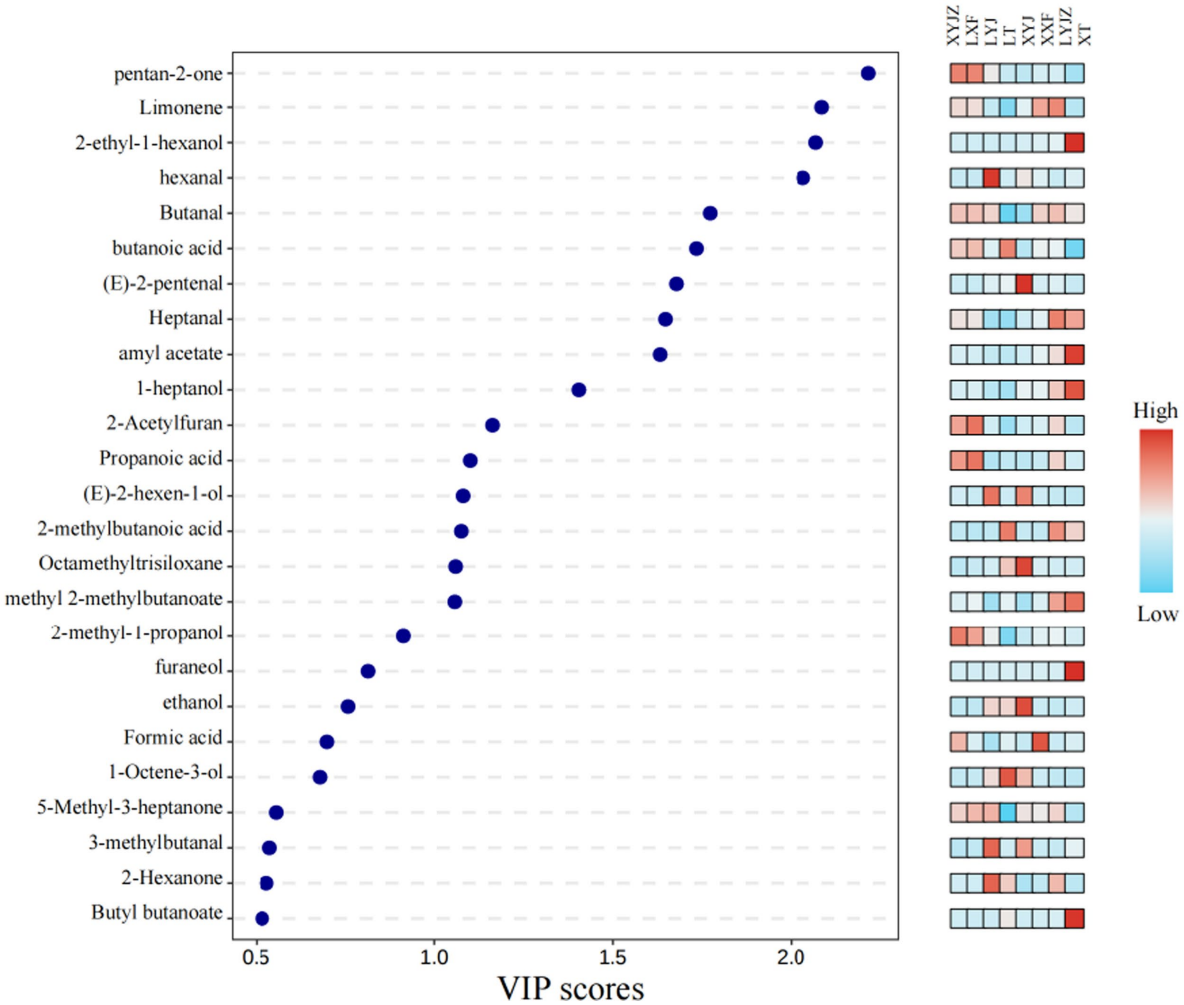
For VIP values.

In the PLS-DA model, the contribution of each variable to classification was quantified based on the VIP (Variable Importance in Projection) values. VOCs with VIP values greater than 1 were selected as potential characteristic flavor compounds (38). As shown in Figure 10, 16 differential VOCs with VIP > 1 were identified:2-pentanone,ethyl hexanoate,2-acetylfuran,butanal, Amyl acetate,heptanal,1-heptanol,1-octen-3-one,1-octen-3-ol,2-methyl-1-propanol, propionic acid, methyl isovalerate, ethanol, 2-ethyl-1-hexanol, methyl butyrate, and(Z)-3-octen-1-ol.

These compounds were likely produced through Maillard reactions, lipid oxidation, and other processes (39), contributing to the unique flavor profile of peanut porridge. The method combining PLS-DA with VIP > 1 has also been successfully used to identify differential VOCs in various processed products, such as different citrus cultivars (40).

## 4 Discussion

This study systematically analyzed the effects of different processing methods on the composition and content of 47 VOCs in peanut porridge. The results revealed that processing methods significantly regulated the relative abundance of key flavor compounds such as alcohols, ketones, aldehydes, and esters, thereby imparting distinct aroma characteristics to the samples. Specifically, the XT (fresh peanut porridge) sample exhibited the highest content of alcohols, particularly 1-heptanol and 1-octen-3-ol, which contributed grassy and mushroom-like fresh notes. In contrast, the XYJ (fresh peanut slurry) sample showed a significant increase in ethanol content, serving as the primary source of alcoholic flavor. Aged peanut samples (LYJ, LYJZ) displayed higher levels of ketones, such as 2-pentanone and 1-octen-3-one, presenting rich nutty, caramel, and roasted aromas. Meanwhile, LXF and XYJZ samples were enriched with esters (e.g., butyl acetate and ethyl propionate), contributing pronounced fruity notes. Additionally, LT and LYJ samples contained higher levels of alkanes and siloxanes (e.g., pentamethylheptane and octamethyltrisiloxane), further enhancing their aroma complexity. These differences indicate that raw material sources and thermal processing conditions can reshape VOC profiles by modulating lipid oxidation, Maillard reactions, and esterification processes, ultimately influencing the sensory attributes of the product.

From a sensory perspective, VOCs are the core substances that construct the overall flavor perception of food, and their types and concentrations directly affect consumers’ perception of odor and taste quality. The study found that aldehydes such as hexanal and heptanal, with their grassy and nutty characteristics, are key contributors to “freshness” and “richness.” Alcohols like 1-octen-3-ol and 2-ethylhexanol impart mushroom and grassy notes, enhancing the natural flavor expression of the product. Esters (e.g., amyl acetate and ethyl propionate) exhibit distinct fruity and creamy aromas, improving the smoothness and pleasantness of the taste. Ketones such as 2-pentanone and furanone present roasted and caramel notes, serving as major sources of flavor intensity in aged peanut porridge. Acids like butyric acid exhibit milky or yogurt-like characteristics, enhancing sensory richness and flavor balance. Given the high sensitivity of the human olfactory system to specific VOCs, even low-concentration compounds can significantly influence the overall aroma profile. Therefore, the differential VOCs among samples not only determine their flavor expression but also largely affect sensory acceptability and perceived product quality. These results provide important insights into the mechanisms by which processing methods shape the flavor of peanut porridge, offering theoretical support for subsequent flavor optimization and standardization.

Furthermore, to ensure the validity and sensory representativeness of the flavor data obtained from electronic nose and GC-IMS techniques in this study, it is necessary to reference relevant research verifying their accuracy. Multiple studies have reported strong consistency between electronic nose/GC-IMS quantitative detection results and human sensory evaluations in terms of aroma characterization and sample classification. For example, Wang et al. (41) combined electronic nose and GC-IMS to analyze the aroma profiles of tea, demonstrating high agreement between principal component analysis results and sensory evaluations, confirming their synergistic effectiveness in aroma differentiation. Yang et al. (22) conducted a triple validation of GC-IMS, electronic tongue/nose data, and manual sensory assessments for various bean pastes, revealing strong correspondence between aroma principal components and sensory descriptors, further supporting the applicability of this combined approach in complex flavor systems. Additionally, Li et al. (42) compared electronic nose, HS-SPME-GC–MS, and sensory panel scores during shrimp paste fermentation, finding high consistency in the trends of key volatiles, confirming the accuracy and stability of electronic sensory techniques in studying dynamic flavor changes. In summary, these findings validate the reliability of electronic nose and GC-IMS in flavor identification and sample classification, further corroborating the scientific rigor of this study’s analytical results. The integration of electronic sensory devices and GC-IMS technology not only overcomes the subjective bias and poor repeatability of traditional sensory evaluations but also offers advantages such as high sensitivity, quantifiable data, and analytical efficiency, making it a crucial supplementary tool for food flavor research and quality control.

From a practical perspective, the findings of this study provide important guidance for quality control and product development of peanut-based foods. On one hand, the identified differential VOCs can serve as key flavor markers for raw material selection, process optimization, flavor monitoring, and product grading, enabling standardized and traceable management. On the other hand, a deeper understanding of how different processing methods influence flavor formation mechanisms can guide enterprises in optimizing formulations and innovating flavors for new products such as instant peanut porridge, peanut beverages, and plant-based functional foods, enhancing market competitiveness and consumer acceptance.

Moreover, while instrumental analysis provides high-throughput, objective, and quantifiable data, it cannot fully replace human perception of aroma and flavor. To further confirm the contribution of identified VOCs to actual flavor perception, sensory validation methods are essential. For instance, GC-O can directly link specific compounds to their corresponding odor perceptions, identifying truly olfactorily active key substances. Sensory evaluation panels can provide human-centered feedback on flavor intensity, balance, and preference. Future research should integrate sensory validation with existing chemical analysis to bridge the gap between “chemical composition—sensory attributes—consumer preference,” thereby enhancing the explanatory power and application value of flavor research and supporting the high-quality, differentiated development of peanut-based foods.

Finally, it should be noted that this study focused on two key stages of peanut porridge processing—the raw slurry state before processing and the cooked final state after processing—without continuous sampling at intermediate time points during heating. Although we successfully revealed the effects of raw material types and thermal processing on volatile flavor composition, key flavor drivers that may transiently form or accumulate during dynamic heating or cooling processes could have been overlooked. This design limitation means the results are more suitable for evaluating flavor differences between pre- and post-processing endpoint samples rather than comprehensively reflecting the temporal evolution and mechanistic pathways of flavor formation. Future studies should adopt time-resolved sampling strategies, combining dynamic tracking and multi-time-point detection to systematically elucidate the evolution patterns and transformation pathways of flavor compounds during the entire heating process, thereby enriching the scientific understanding of flavor formation mechanisms.

## 5 Conclusion

In this study, GC-IMS technology, along with electronic tongue and electronic nose techniques, was employed in combination with chemometric methods such as PCA and PLS-DA to analyze the differences and variations in flavor compounds among differently processed peanut porridge samples. Aged peanut porridge exhibited a complex and intense aroma with roasted, caramel, and nutty characteristics, while fresh peanut porridge retained a fresher, more natural aroma, preserving the original flavor of the raw materials and offering a light and refreshing taste. Electronic tongue and electronic nose techniques effectively distinguished peanut porridge samples subjected to different treatments. Using GC-IMS, 47 VOCs were identified, and 16 differential volatile flavor compounds were screened based on VIP > 1, primarily consisting of ketones, aldehydes, and alcohols. The combined application of electronic tongue, electronic nose, and GC-IMS offers rapid, sensitive, and comprehensive advantages, making it highly valuable for flavor analysis, process optimization, and quality control in food science. The case study of peanut porridge demonstrates that this integrated approach can precisely reveal the influence of raw materials and processing on flavor, providing scientific support for the modern production of traditional foods. The relative content of various volatile compounds exhibited dynamic changes depending on the processing method, likely due to factors such as Maillard reactions, lipid oxidation, or thermal degradation. Based on PLS-DA analysis, VIP values were used to identify the differential volatile compounds in peanut porridge. PCA results showed that the cumulative contribution rate of the first two principal components reached 66.7%, indicating that these characteristic volatile components effectively differentiated the peanut porridge samples. Comprehensively characterizing the differences in volatile compounds among peanut porridge samples processed under different conditions helps clarify the impact of various treatments on the final flavor profile, providing data support for quality control and process optimization. This study offers a theoretical basis for the targeted flavor improvement and standardized processing of peanut-based products. However, further validation through GC-O or sensory omics is needed to assess compound thresholds and their synergistic effects.

## Data Availability

The original contributions presented in the study are included in the article/supplementary material, further inquiries can be directed to the corresponding author.
